# Development and application of one-step multiplex Real-Time PCR for detection of three main pathogens associated with bovine neonatal diarrhea

**DOI:** 10.3389/fcimb.2024.1367385

**Published:** 2024-04-02

**Authors:** Chaonan Wang, Fang Wang, Jitao Chang, Zhigang Jiang, Yuxin Han, Meixi Wang, Bo Jing, Aiyun Zhao, Xin Yin

**Affiliations:** ^1^ State Key Laboratory for Animal Disease Control and Prevention, Harbin Veterinary Research Institute, Chinese Academy of Agricultural Sciences, Harbin, China; ^2^ College of Animal Science and Technology, Tarim University, Alar, Xinjiang, China; ^3^ Institute of Western Agriculture, The Chinese Academy of Agricultural Sciences, Changji, China

**Keywords:** neonatal calf diarrhea, bovine rotavirus, bovine coronavirus, *Escherichia coli* k99, real-time PCR

## Abstract

**Introduction:**

Neonatal calf diarrhea (NCD) is one of the most common diseases in calves, causing huge economic and productivity losses to the bovine industry worldwide. The main pathogens include bovine rotavirus (BRV), bovine coronavirus (BCoV), and Enterotoxigenic Escherichia coli (ETEC) K99. Since multiple infectious agents can be involved in calf diarrhea, detecting each causative agent by traditional methods is laborious and expensive.

**Methods:**

In this study, we developed a one-step multiplex Real-Time PCR assay to simultaneously detect BRV, BCoV, and E. coli K99+. The assay performance on field samples was evaluated on 1100 rectal swabs of diseased cattle with diarrhea symptoms and compared with the conventional gel-based RT-PCR assay detect BRV, BCoV, and E. coli K99+.

**Results:**

The established assay could specifically detect the target pathogens without cross-reactivity with other pathogens. A single real-time PCR can detect ~1 copy/µL for each pathogen, and multiplex real-time PCR has a detection limit of 10 copies/µL. Reproducibility as measured by standard deviation and coefficient of variation were desirable. The triple real-time PCR method established in this study was compared with gel-based PT-PCR. Both methods are reasonably consistent, while the real-time PCR assay was more sensitive and could rapidly distinguish these three pathogens in one tube. Analysis of surveillance data showed that BRV and BCoV are major enteric viral pathogens accounting for calves’ diarrhea in China.

**Discussion:**

The established assay has excellent specificity and sensitivity and was suitable for clinical application. The robustness and high-throughput performance of the developed assay make it a powerful tool in diagnostic applications and calf diarrhea research. ​

## Introduction

Neonatal calf diarrhea (NCD) contributes significantly to calf mortality and morbidity worldwide. As reported, during the first 30 days after birth, the case fatality risk caused by NCD is approximately 5% (Svensson et al., 2006; Windeyer et al., 2014). NCD is mainly caused by infection with viruses, bacteria, and parasites. Of these, bovine rotavirus (BRV), bovine coronavirus (BCoV), and enterotoxigenic *Escherichia coli* K99 (ETEC) are the most frequently identified causative factors (Acres, 1985; Krogh and Henriksen, 1985; Tzipori, 1985; Snodgrass et al., 1986). These pathogens are often mixed and lead to several complications. Thus, early diagnosis and treatment facilitate timely management and control of NCD.

BRV is believed to be the leading cause of viral diarrhea disease of newborn calves. BRV is a non-enveloped and double-stranded RNA virus that belongs to the genus *Rotavirus* in the family *Reoviridae*. Its genome comprises 11 double-stranded RNA segments that encode six structural proteins (VP1−VP4, VP6−VP7) and six nonstructural proteins (NSP1−NSP6). Based on the antigenicity of the middle VP6 capsid antigen, rotavirus is classified into at least 10 groups (A-J) (Bányai et al., 2017). Among which, group A rotavirus cause disease in both humans and animals. Group A rotaviruses can be further divided into P and G types based on the genetic and antigenic characteristics of VP4 and VP7, respectively. In cattle, G6, G8, and G10 strains appear to be the dominant strain circulating in the herd (Snodgrass et al., 1990; Ciarlet et al., 1997; Rao et al., 2000). As reported, the pooled prevalence of BRV in cattle was approximately 46% in China (Chen et al., 2022).

In addition to BRV, BCoV is frequently identified from the calves with viral diarrhea disease. BCoV is a single-stranded positive-sense RNA virus with a lipid envelope that belongs to the genus *Beta-coronavirus* within the *Coronaviridae* family (Pfefferle et al., 2009; Yang and Leibowitz, 2015; Oma et al., 2016). There are 13 open reading frames (ORFs) in the approximately 31 kb-long RNA genome, bordered by 5′ caps and 3′ polyadenylated tails (Chouljenko et al., 2001). The nucleocapsid protein (N), membrane protein (M), minor capsid protein (E), spike protein (S), and hemagglutinin esterase protein (HE) are the five structural proteins inducing antibodies. In addition to severe diarrhea in newborn calves, BCoV infection causes respiratory illness in calves and feedlot cattle, and winter dysentery in adult cattle (Gomez and Weese, 2017). However, less systematic studies have been conducted to evaluate the prevalence of BCoV infection in China (Geng et al., 2023). Infections with either BRV or BCoV, usually result in intestinal injury and immune suppression. This could increase the likelihood of infection with *E. coli* K99^+^.

Since NCD is usually caused by mixed infections with multiple pathogens and has similar clinical symptoms, they are not easy to differentiate. In the complex chain of epidemiologic and therapeutic measures to prevent outbreaks, there is a need for rapid and sensitive diagnostic tools for early differential diagnosis of mixed infection. The traditional detection methods (e.g. virus isolation, ELISA, PCR, etc.) have some limitations (Decaro et al., 2008; Mijatovic-Rustempasic et al., 2013; Jesser and Levy, 2020). Therefore, single, and multiplex real-time PCR assays have been developed for the rapid detection of pathogens associated with NCD in the past years (West et al., 2007; Decaro et al., 2008; Cho et al., 2010; Mijatovic-Rustempasic et al., 2013). Single detection methods require separate amplification of the pathogens, which is a cumbersome and time-consuming process. Although, BCoV, BRV, and *E. coli* K99^+^ are reported to be the major cause of NCD (Brunauer et al., 2021). There are no multiplex methods that place these three pathogens in the same reaction for detection currently.

In this study, a multiplex real-time PCR assay based on TaqMan technology was developed for the rapid and sensitive diagnosis of BCoV, BRV, and *E. coli* K99^+^ infection and accurate quantification of nucleic acid in clinical samples. The method enabled the simultaneous resolution by using of three sets of specific primers and probes in a single reaction mixture. The assay performance was verified by the evaluation of specificity, sensitivity, reproducibility, and dynamic range. Consequently, this method can be effectively used in diagnostic, surveillance, and epidemiologic studies of NCD caused by BRV, BCoV, and *E. coli* K99^+^.

## Materials and methods

### Primer and probe design

All available complete sequences of BRV (forty clinical isolates), BCoV(forty-one clinical isolates), and *E. coli* K99^+^ (eighteen clinical isolates) were retrieved from GenBank. The NSP5 gene sequences of BRV, the N gene sequences of BCoV and the K99(F5)pili sequences of *E. coli* K99^+^ were aligned with DNASTAR software for identifying the highly conserved regions of each gene. Primers and probes are then designed to target the conserved regions via the Primer Premier 5 program. Upon the filtration based on the design criteria such as hairpin configurations and primer dimer formation. Three pairs of primers and probes were picked for the synthesis by Comate Bioscience Company (Jilin, China), and the detailed information is shown in **Table 1**.

**Table 1:** Primers and probes used in this study.

Pathogens	Primer or probes	Sequence (5’-3’)	Position
BRV	BRV-NSP5-U	TGCTTCAAACGATCCACTCAC	216--236
	BRV-NSP5-L2	CCACTTGATCGCACCCAAC	328-346
	BRV-NSP5-P2	FAM-TTAACTGCATTCGATCTAATCG-BHQ1	245
BCoV	BCoV N476F	CTGACATTCTCGATCGGGAC	476-495
	BCoV N570R	TTAGGAGCAGACCTTCCTGAG	570-590
	BCoV N502P	VIC-AGCGATGAGGCTATTCCGACTAGG-BHQ1	502
*E. coli* K99^+^	K99-U1	TCGTACATCAACTATAGATC	210-229
	K99-L2	AGAACCAGACCAGTCAATACG	325-345
	K99-P2	CY5-GGCTGCTATTAGTGGTCATGGCACT-BHQ1	237-261

### Viruses, bacterial and cell cultures

BRV G6 strain (C7-3), BRV G10 strain (HM26), BCoV strain (HM-XC), Bovine Viral Diarrhea Virus (BVDV NADL strain), Bovine Enterovirus (BEV strain BHM26 and BJ50) (Li et al., 2012), Bovine Parvovirus (BPV-1 strain HADEN) (Chang et al., 2019), *Salmonella enterica (S. enterica)* strain (FH10) (Lee et al., 2009) *Clostridium perfringens (C. perfringens)* type A strain CVCC C57–8 was purchased from the China Institute of Veterinary Drug Control (Beijing, China), and *E. coli* K99+ strain HLJ23 isolated from calf diarrhea. TCID50 of the BcoV, BEV, BPV and BRV were determined in HRT-18G (ATCC, CRL-3609), BHK-21 cell, primary bovine testicular cell and Marc-145 (a clone of the MA-104 cell line, ATCC CRL-2378), respectively. The colony-forming units (CFU) of E. coli K99+ strain was quantified by the traditional plate colony counting method. Both viruses and bacteria mentioned above were sequenced.

DNA/RNA was extracted from cell cultures, bacteria, and clinical samples according to the manufacturer’s instructions of the AxyPrep body fluid viral DNA/RNA miniprep kit (Axygen Biosciences, USA). The obtained nucleic acids are stored at -80°C until needed. To obtain the standard plasmid, the target fragments of BRV, BcoV and *E. coli* K99^+^ were amplified and cloned into the pMD18-T vector (Takara, China). The standard plasmids were then transformed into DH5α chemically competent cells, and the plasmids were extracted using the GeneJET Plasmid Miniprep Kit (ThermoFisher Scientific,USA). The concentration of the purified plasmid DNA was determined by measuring the optical density at 260 nm (OD _260_) with a Nanodrop One (ThermoFisher Scientific, USA)

The copy number of the extracted plasmids was calculated according to the following formula.


$$\text{Plasmid copies}/µ\text{L}=\frac{\left(6.02\times 10^{23}\right)\times \left(\text{X ng}/µ\text{L}\times 10^{-9}\right)}{\text{Plasmid length }\left(\text{bp}\right)\times 660}$$

Single real-time PCR was performed using serial 10-fold dilutions of the plasmid from 10^7^ to 1 copies/µL, and the plasmid from 10^7^ to 10^1^ copies/µL were selected to construct the standard curve. Three standard curves were constructed, and corresponding equations were determined by taking amplification efficiency and correlation coefficients into account.

### Optimization of the single and multiplex TaqMan real-time PCR assays

Single real-time PCR was performed using the HiScript II U^+^ One Step qRT-PCR Probe Kit from Vazyme. The total volume of the PCR system was set as 10 µL, including 2× One Step U^+^ MIX, One Step U^+^ Enzyme MIX, and 50× ROX Reference Dye 2, the amount of the primer addition was optimized from 0.1 to 0.8 µL, while the amount of the probe addition was optimized from 0.1 to 0.6 µL, which were both at a final concentration of 10 µM. Amplification was carried out using the following program: 55°C 15 min, 95°C, 30 s, 45 cycles of 95°C 10 s, annealing temperature was optimized between 54°C and 60°C. Amplification with Quant Studio™ Design & Analysis Software. Signals were automatically collected at the end of each cycle.

Multiplex real-time PCR was established based on three single methods by adjusting the reaction system and conditions to reduce interference among three different fluorescent molecules and to increase amplification efficiency. The final concentration for the primer and probe was both set as 10 µM, and the amount of primer additions was optimized between 0.1 µL and 0.5 µL, while the amount of probe additions was optimized from 0.1 µL to 0.4 µL. The reaction temperature was optimized between 55°C and 65°C.

### Sensitivity and specificity of the multiplex real-time PCR assay

Multiplex real-time PCR was as performed using the HiScript II U^+^ One Step qRT-PCR Probe Kit from Vazyme. A 10-fold gradient dilution of 10^8^−10^1^ copies/µL of the standard plasmids served as the template. Multiplex real-time PCR was conducted in tube, with three replicates assigned to each reaction to examine the detection limits of multiplex reactions. The primers and probes used in this study were tested for specificity. To avoid false positives caused by other pathogens that may be present in the assay, the multiplex real-time PCR detection assay was used to detect BVDV, BEV, BPV, *S. enterica*, and *C. perfringens* and RNA-free H_2_O as negative control.

### Repeatability test

Standard plasmids with four different copy numbers (10^3^−10^6^) were used for the repeatability test of the real-time PCR assay. Three replicate experiments were set up in both intra- and inter-groups. The standard deviation and coefficient of variation were calculated for the three reactions.

### Examination of clinical fecal samples using real-time PCR

A total of 1100 rectal swabs were collected from cattle in different regions including Heilongjiang Province, Inner Mongolia Autonomous Region, Ningxia Hui Autonomous Region, Jilin Province, Henan Province, and Jiangsu Province, etc. The majority of calves included in the samples showed diarrheic symptoms. The swabs were preserved at -80°C until needed.

## Results

### Primers and probe for real-time PCR

By comparison and analysis of the retrieved sequences from GenBank, we found that the segment 11, which encodes NSP5 protein is most conserved among the 11 segments of BRV genome. Thus, the fragment ranging from 216 bp to 346 bp in segment 11 gene was selected as a target for primer and probe design. The N gene is the most conserved segment of BcoV, and we chose the 476-590 bases at the 5’ end of the N gene to design primers and probes, and the amplified fragment length was 115 bp. In addition, the *E. coli* K99^+^ (F5) pilis gene is often used as a target for detecting *E. coli* infections, and we chose the most conserved region 210−345 bases in *E. coli* K99^+^ (F5) pilis gene for the design. The primers and probes designed for real-time PCR were listed in **Table 1**. Theoretically, these primer/probe sets could recognize all BRV, BcoV, and *E. coli* K99^+^ strains prevalent in the herd. To establish a standard curve, each of the three amplicon fragments was cloned into the pMD18-T vector and used as a standard plasmid for further use.

### Specificity analysis of primer/probe sets

**Figure 1:**
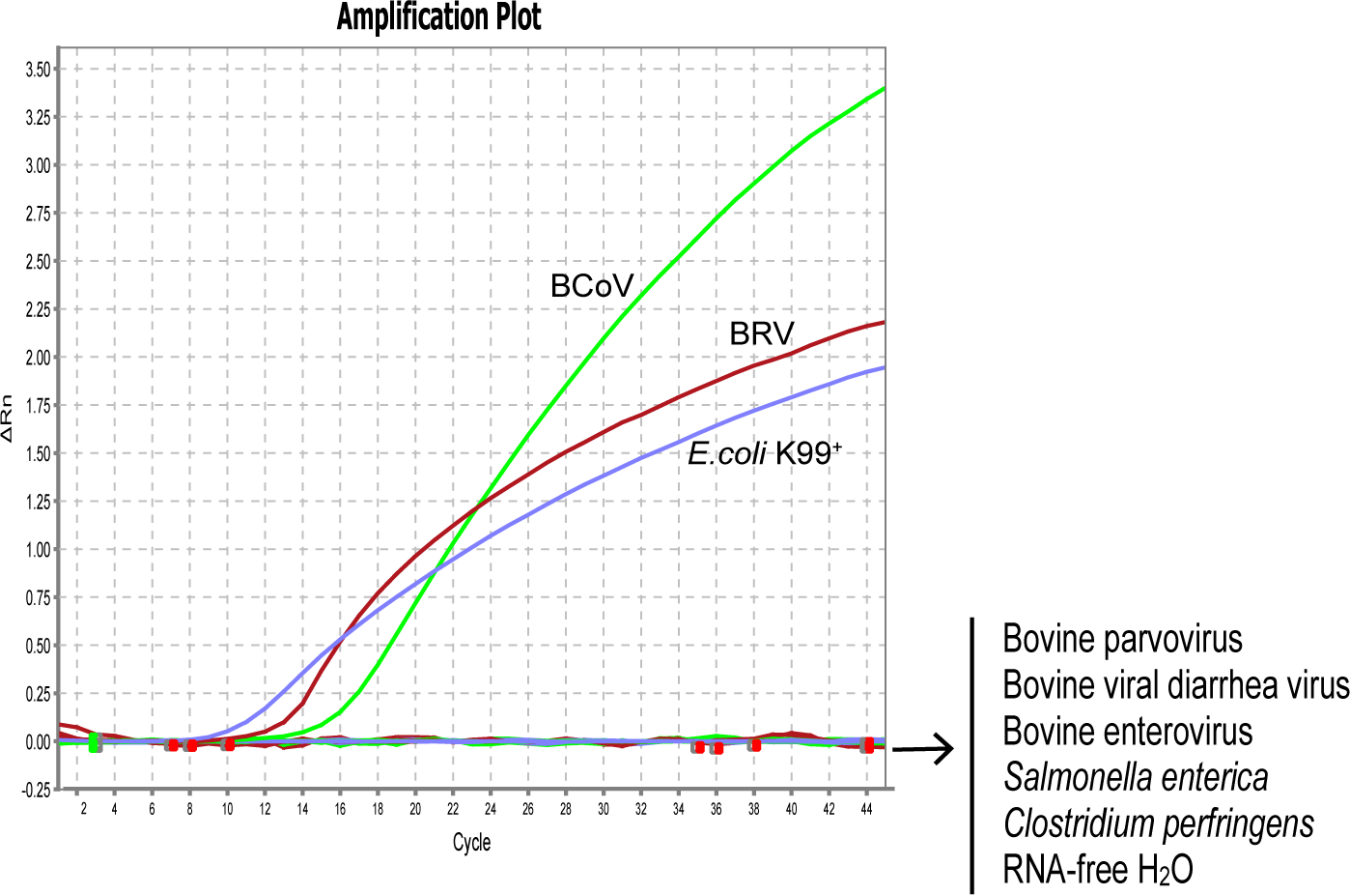
Analytical specificity of the primer/probe sets used in the one-step real-time RT-PCR assay: Amplification curves represent samples positive for BRV, BCoV, and *E. coli* K99^+^ detected by the real-time Negative samples include Bovine viral diarrhea virus, Bovine enterovirus, Bovine parvovirus, *Salmonella enterica, Clostridium perfringens*, and RNA-free H_2_O. Equal amount of isolated viral/bacterial genome were mixed for the assay. The non-detected samples are labeled on the right side of the figure.

To evaluate the specificity of our designed primers and probes, we selected 1×10^6^ copies/µL of standard plasmids harboring the specific fragments of BRV, BcoV, and *E. coli* K99^+^ for the assay. As expected, all the primers and probes only bind to their target regions without any cross-reaction. To further verify the specificity of the designed primer/probe sets, the genomes were extracted from the strains of BRV, BcoV, BVDV, BEV, BPV, *E. coli* K99^+^, *S. enterica*, and *C. perfringens* for assessment. Consistently, the designed primers and probes for detecting BRV, BcoV, and *E. coli* K99^+^ could specifically recognize the genome isolated from BRV, BcoV, and *E. coli* K99^+^. There were no signals detected for other pathogens, confirming that the real-time PCR assay is specific for BRV, BcoV, and *E. coli* K99^+^. (**Figure 1**)

### Optimized single real-time PCR

**Figure 2:**
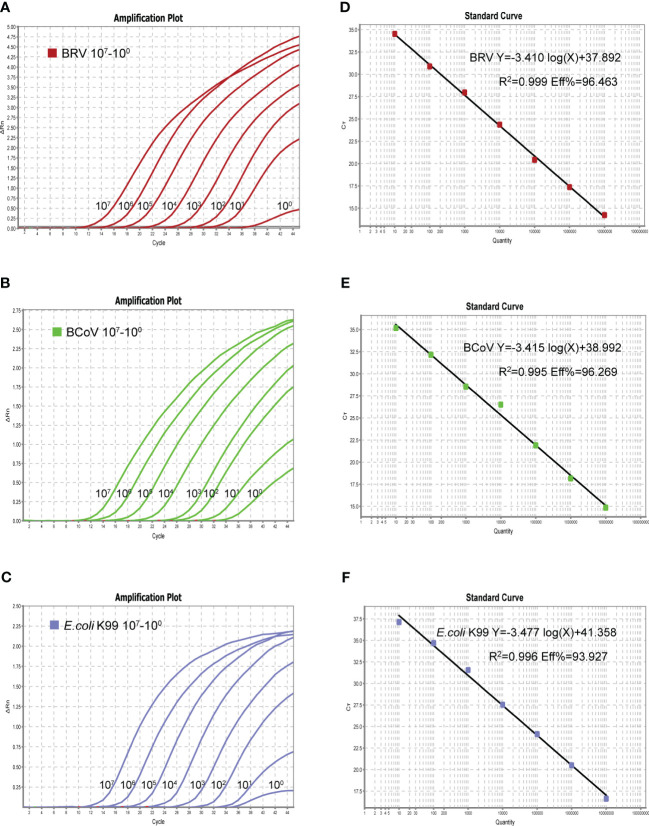
The amplification curves (X-axis: Cycle, Y-axis: △Rn) of the plasmid standards are shown in left panel **(A–C)**. The plasmid was diluted in a concentration range of 10^7^-1 copies/µL. Standard curves of 10^7^-10^1^ copies/µL plasmid standards for BRV, BCoV, and *E. coli* K99^+^ **(D–F)**. BRV: Y=−3.410log(X)+37.892 R^2 ^= 0.999 Eff%=96.463, BCoV: Y=−3.415log(X)+38.992 R^2 ^= 0.995 Eff%=96.269, K99: Y=−3.477log(X)+ 41.358 R^2 ^= 0.996 Eff%=93.927.

Upon confirmation of the specificity of the designed primers and probes used in this study, a single real-time PCR assay was first generated for individual pathogen. The optimized reaction system for the single assay was 10 µL: 5 µL of 2× One Step U^+^ MIX, 0.5 µL of One Step U^+^ Enzyme MIX, 0.2 µL of 50× ROX Reference Dye 2, 0.2 µL of each primer (10 µM), 0.3 µL of each probe (10 µM), 2.6 µL of Rnase-free dd H_2_O and Template 1 µL. The single assay can detect a minimum of 1 copy of the standard plasmid, as shown in **Figure 2** (A-C left panel). The real-time PCR amplifications of each standard plasmid DNA showed amplification plots corresponding to mean Ct values of 10.343−40.853 for BRV, 12.346−35.181 for BcoV, and 12.622−36.709 for *E. coli* K99^+^ (**Figure 2**). As the linear range includes 8 orders of magnitude (from 1 to 10^7^ copies of the standard plasmid), we chose 10^1^−10^7^ copies to establish the standard equation (**Figures 2A–C**). The slope of the standard curve, correlation coefficient (R^2^), and amplification efficiency (Eff%) were calculated as follows −3.140, 0.999, and 96.463% for BRV; −3.415, 0.995, and 96.269% for BcoV; −3.477, 0.996, and 93.927% for *E. coli* K99^+^. (**Figures 2D–F**). Collectively, these results demonstrated that the optimized single real-time PCR were sensitive for the detection of BRV, BcoV, and *E. coli* K99^+^ individually.

### Optimization of the multiplex reaction system

**Figure 3:**
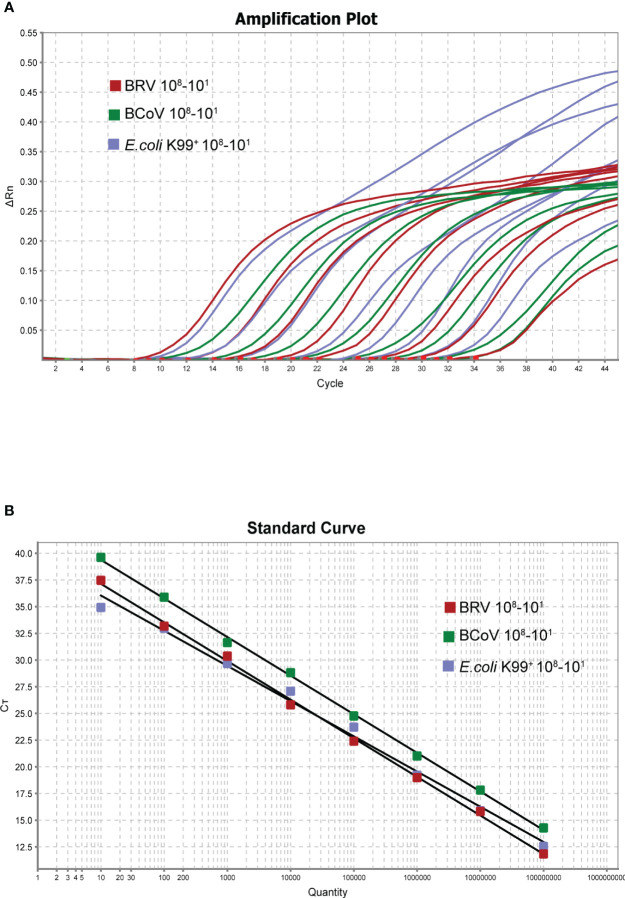
Amplification curve of triple reaction (X-axis: number of cycles, Y-axis: △Rn) **(A)**. The copy number of each viral plasmid ranged from 10^8^-10^1^ copies/µL. Standard curve (X-axis: copy number, Y-axis: CT value) **(B)**. BRV: Y=−3.616 log(X)+40.756 R^2 ^= 0.998 Eff%=89.028, BCoV: Y=−3.427log(X)+41.866. R^2 ^= 0.995 Eff%=95.810, *E*. *coli* K99^+^: Y=−3.298log(X)+39.343 R^2 ^= 0.993 Eff%=101.021.

Following the optimization of the single real-time PCR for each pathogen, the multiplex real-time PCR was developed through multiplex rounds of optimization. We found that a good amplification effect can be achieved when the total reaction system is 20 µL, and the addition of each component was multiplied twice compared to the single system, with optimized primer and probe additions: 0.1 µL NSP5-U/NSP5-L2 primer set (10 µM), 0.1 µL BRV-NSP5-P2 (10 µM), 0. 1 µL N476F/N570R primer set (10 µM), 0.1 µL BcoV N502P (10 µM), 0. 2 µL K99-U1/K99-L2 primer set (10 µM), 0. 2 µL K99-P2 (10 µM) The annealing temperature was optimized to be 55°C. Good correlation coefficients and amplification efficiencies for the standard curves were observed when the total volume was 20 µL (**Figures 3A, B**) BRV (R^2 ^= 0.998 Eff%=89.028), BcoV (R^2 ^= 0.995 Eff%=95.810), *E. coli* K99^+^. (R^2 ^= 0.993 Eff%=101.021). The optimized multiple-reaction system was able to identify specific pathogens effectively, and the correlation coefficients of the three pathogens were able to reach more than 0.99. The amplification efficiencies of both BcoV and *E. coli* K99^+^ were excellent, although the amplification efficiency of BRV was slightly lower. Taken together, after the optimization, the multiplex real-time PCR was established for further assessment.

### Sensitivity and limit of detection of real-time PCR

By using the 10-fold serial diluted plasmid standards, we further validated the amplification efficiency and detection limits. After extensive experiments, we found that the single real-time PCR can detect all three pathogens down to 1 copy/µL (**Figures 2A–C**), while the detection limit of multiplex real-time PCR only can reach 10 copies/µL (**Figure 3A**). To further verify the sensitivity and detection limits of the multiplex assay, the cultured viruses with specific titers were used for the quantification. The minimum value for detection of BRV in the multiplex real-time PCR assay was 0.5 TCID_50_ (50% tissue culture infected dose) and the minimum value for detection of BcoV was 0.14 TCID_50_. For *E. coli* K99^+^, 0.7433 CFU can be detected. Furthermore, we defined cutoff values for these three pathogens in the multiplex assay. The cutoff CT value for BRV positivity was defined at 36, which means the sample with a CT value less than or equal to 36 (≤ 36) was considered positive, but higher than 36(>36) is negative. The cutoff CT value for BcoV and *E. coli* K99^+^ was 36 and 35, respectively.

### Repeatability of the multiplex real-time PCR

To evaluate the reproducibility of the multiplex real-time PCR, the standard plasmids at the concentrations of 1×10^6^, 1×10^5^, 1×10^4,^ and 1×10^3^ copies/µL were used. As a result, the coefficients of variation of the intra and inter-assay ranged from 0.053% to 1.275% and 0.270% to 3.319%, respectively (**Table 2**), indicating good repeatability of the assay.

**Table 2:** Repeatability of the multiplex real-time PCR.

Target	Templates (copies/µL)	Intra-assay		Inter-assay	
		$\bar{X}\pm SD$	CV(%)	$\bar{X}\pm SD$	CV(%)
BCoV	10^6^	22.282±0.094	0.422	22.438±0.327	1.457
	10^5^	26.130±0.141	0.539	25.905±0.731	2.822
	10^4^	30.709±0.233	0.759	31.001±0.687	2.217
	10^3^	32.938±0.310	0.940	33.360±0.393	1.177
BRV	10^6^	18.963±0.037	0.195	18.449±0.571	3.096
	10^5^	22.410±0.105	0.469	21.728±0.721	3.319
	10^4^	25.937±0.119	0.460	25.988±0.518	1.993
	10^3^	30.478±0.205	0.672	30.347±0.774	2.550
*E. coli* K99^+^	10^6^	19.120±0.063	0.328	18.495±0.095	0.514
	10^5^	23.693±0.013	0.053	22.967±0.223	0.971
	10^4^	26.735±0.341	1.275	26.913±0.073	0.271
	10^3^	29.323±0.251	0.854	30.278±0.833	2.752

$\bar{X}$ , average; SD, standard deviation; CV, coefficient of variation. and mean, Ct value was calculated from three independent wells for triplex real-time PCR run.

### The surveillance of NCD cases in China by using the established multiplex assay

**Figure 4:**
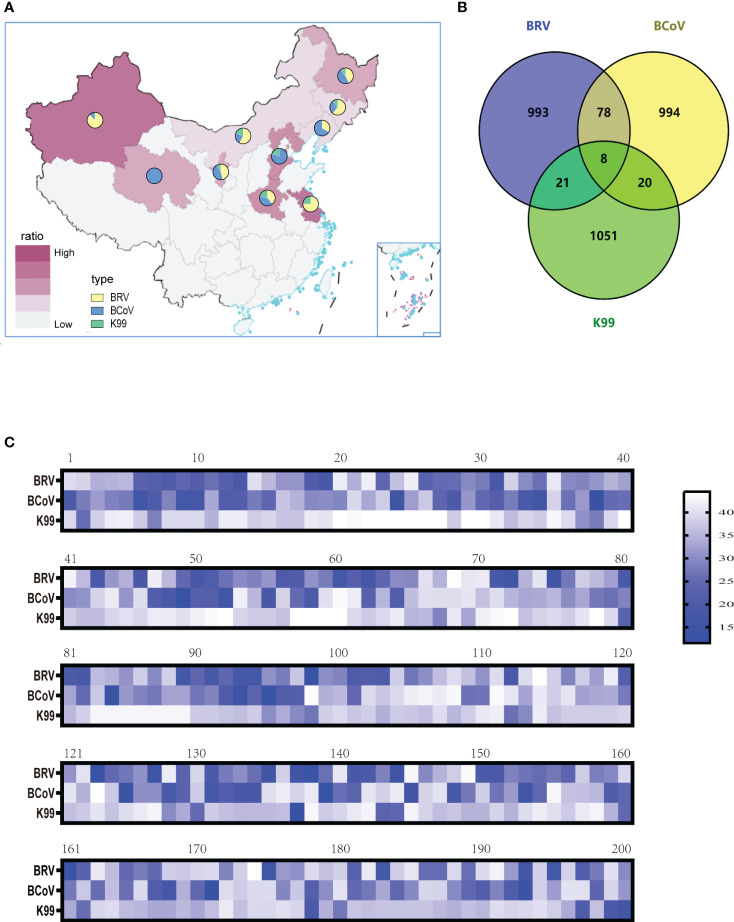
Clinical sample detection: **(A)** BRV, BCoV, and *E*. *coli* K99^+^ infection rates in ten provinces and analysis of the proportions of the three pathogens **(B)**The mix-infection incidence among BRV, BCoV, and *E. coli* K99^+^. **(C)** Selection of 200 clinical samples to analyze the level of pathogen contained (CT value).

To evaluate the optimized assay in clinical application, 1100 rectal swabs of NCD cases were collected in 10 provinces as shown in **Table 3**, the average positive rate for BRV, BcoV, and *E. coli* K99^+^ was 22.64%, 22.00%, and 5.82%, respectively. Further analysis found that the highest positive rate of BRV(58.33%) was observed in Xinjiang region, while Qinghai had the highest positive rate of BcoV (50%) (**Figure 4A**). However, the overall infection rate of *E. coli* K99^+^ was low. In Qinghai, only BcoV infection was detected, while in the other 9 provinces, at least two pathogens were tested positive. Notably, 792/1100 samples were collected from Heilongjiang province, the analysis showed that both BRV and BcoV infections in calves was highly prevalence, which accounted for the occurrence of NCD cases in Heilongjiang province. In addition, we analyzed the co-infection rate in the detected samples, and found that the co-infection of BRV and BcoV was higher at a rate of 7.1% (78/1100), while the co-infection of BRV and *E. coli* K99^+^ as well as BcoV and *E. coli* K99^+^ was similar at 1.9% (21/1100), and 1.8% (20/1100), respectively. Only 8 out of 1100 samples were mix-infected with these three pathogens (**Figure 4B** and **Table 4**). We also picked 200 samples to analyze the CT values of BRV, BcoV, and *E. coli* K99^+^ detected via the multiplex PCR assay (**Figure 4C**), we found that the CT value for BRV and BcoV were lower, compared to that for *E. coli* K99^+^, further confirming that the co-infection of BRV and BcoV occurs frequently in the calves with diarrhea.

**Table 3:** Sample test results statistics.

Provinces	Prevalence of BRV infection	Prevalence of BCoV infection	Prevalence of *E. coli* K99^+^ infection	Total samples
Heilong jiang	(181/792) 22.85%	(197/792) 24.87%	(43/792) 5.43%	792
Inner Mongoria	(36/177) 20.34%	(15/177) 8.48%	(12/177) 6.78%	177
Jilin	(6/25) 24.00%	(5/44) 11.36%	(1/25) 4.00%	25
Henan	(9/38) 23.69%	(9/38) 23.69%	(5/38) 13.16%	38
Ningxia	(6/25) 24.00%	(6/25) 24.00%	(1/25)4.00%	25
Jiangsu	(3/6) 50.00%	(0/6) 0%	(1/6) 16.67%	6
Qinghai	(0/6) 0%	(3/6) 50.00%	(0/6) 0%	6
Liaoning	(1/10) 10%	(2/10) 20%	(0/10) 0%	10
Hebei	(0/9) 0%	(4/9) 44.44%	(1/9) 11.11%	9
Xinjiang	(7/12) 58.33%	(1/12) 8.33%	(0/12) 0%	12

**Table 4:** Analysis of mix-infections.

pathogens	BRV	BCoV	*E. coli* K99^+^
Total infection rate	(249/1100)22.64%	(242/1100)22.00%	(64/1100)5.82%
Separate infection rate	(142/1100)12.90%	(136/1100)12.36%	(15/1100)1.36%
Double infection rate	(99/1100)9.00%	(98/1100)8.90%	(41/1100)3.73%
Triple infection rate	(8/1100)0.73%	(8/1100)0.73%	(8/1100)0.73%

To further confirm the results obtained from the multiplex assay, we performed the conventional gel-based RT-PCR assay in parallel for comparison. In 1100 samples, the multiplex PCR assay could detect 249 BRV positive samples, while gel-based RT-PCR only detected BRV 230 samples (**Table 5**). For BcoV detection, 242 samples were positive in the multiplex assay, while only 226 samples were positive in the gel-based RT-PCR assay. Similar results were also observed for *E. coli* K99^+^ (64 positive in the multiplex assay and 41 positive in the gel-based RT-PCR assay). These results demonstrated that our established multiplex PCR assay was more sensitive than the conventional gel-based RT-PCR assay for the clinical application.

**Table 5:** Comparative analysis of bovine clinical samples using real-time PCR and gel-based RT-PCR assays.

	BRV No. (%)	BCoV No. (%)	*E. coli* K99^+^ No. (%)
Real-time PCR			
Positive	249 (22.64)	242 (22.00)	64 (5.82)
Negative	851 (77.36)	858 (78.00)	1036 (94.18)
Gel-based RT-PCR			
Positive	230 (20.91)	226 (20.55)	41 (3.73)
Negative	870 (79.09)	874 (79.45)	1059 (96.27)
Total	1100	1100	1100

## Discussion

NCD is a significant global issue in the cattle industry, which deeply impact calves’ growth rate and lifetime productivity. As reported, it is a muti-factorial disease, involving both infectious and non-infectious factors (Oguejiofor et al., 2019). The high probability of mixed infections of these three pathogens makes the rapid detection of these pathogens of great importance. Among these etiological agents that cause calf diarrhea, BCoV and BRV account for about 27%–36% (Cho and Yoon, 2014), which is consistent with our detection results. Moreover, the main pathogenic bacteria causing NCD are reportedly enterotoxigenic *E.coli* K99^+^ (Cho and Yoon, 2014). However, in our detected clinical samples, the positive rate of *E.coli* K99^+^ is lower (0% to 16.67%), which might be due to the antibiotics usage in the clinical management prior to the detection.

At present, several diagnostic assays have been reported to individually detect BCoV, BRV, and *E. coli* K99^+^. A sensitive quantitative real-time PCR method for detecting BCoV in fecal samples could detect 10^2^ copies/reaction (Decaro et al., 2008). In addition, one-step real-time reverse transcription-PCR assay was previously established to detect rotavirus A approximately 1 genome copy per reaction (Mijatovic-Rustempasic et al., 2013). In our single real-time PCR detection assay, through a series of optimizations, we could achieve a sensitivity of 1 copy/µL for the detection of BCoV and BCoV. More recently, a multiplex real-time PCR method was also reported by dividing six bovine diarrhea viruses into two systems for detection (Meng et al., 2023). However, the detection limits only reached to 16.4 copies/µL and 18.2 copies/µL for BRV and BCoV, respectively. In our study, the sensitivity was up to 10 copies/µL for all 3 detected pathogens, suggesting that our developed assay is more robust for clinical detection. In addition, different from previous reports using the segment 8 as the target for BRV detection, we chose the segment 11 gene in our single-weighted method. Similar to our assay, two panels of multiplex real-time PCR assays, one for viral agents (BCoV and BRV) and the other for bacterial/protozoan agents (*E. coli* K99^+^, *Salmonella*, and *Cryptosporidium*) were recently established (Cho et al., 2010). This assay can effectively perform multiplex detection of diarrhea pathogens, however, the separation of viruses and bacteria into two test tubes is still not convenient enough in the clinical application. In our study, BCoV, BRV, and *E. coli* K99^+^, the major causes of diarrhea were built into one multiplex assay, Pathogens whose genetic material is DNA and RNA can be tested in a single reaction tube without the need for an additional reverse transcription step. The performance of this assay showed high sensitivity and specificity. More importantly, the detection results can be acquired within less than 2 hours.

In our clinical study, we found that calf diarrhea is widespread throughout China, especially in northeastern China. In the 1100 feces collected from calves with diarrhea in 10 provinces (**Table 3**). BRV prevalence exceeds 20% in seven provinces, and six provinces had BCoV prevalence rates above 20%. It appears that BRV remained the main etiological agents causing NCD in China, which is consistent with previous reports (Cheng et al., 2021). Intriguingly, the *E. coli* K99^+^ detection rate in the herd is lower, which may be due to the current widespread use of antibiotics. In addition, the mixed infections occurred frequently, especially for BCoV and BRV(**Table 4**). However, currently, there are no commercial vaccines against BCoV and BRV available in China. Therefore, the further surveillance of BCoV and BRV circulating in China and the effective vaccine development are urgent.

The efficacy of the established multiplex real-time PCR assay was evaluated by analysis of 1100 rectal swab samples in comparison to the gel-based RT-PCR assay (**Table 5**). In general, the developed multiplex real-time PCR assay is more sensitive than the conventional gel-based RT-PCR assay. Some of the samples tested negative by gel-based RT-PCR were found to be positive by real-time PCR. While none of the samples tested negative by real-time PCR was found positive by the gel-based method. Based on the above conjecture, we selected 200 samples for statistical analysis of CT values (**Figure 4C**), Indeed, the positive samples confirmed by real-time PCR assay rather than conventional gel-based RT-PCR assay had higher CT values. Thus, the established multiplex PCR assay would provide the conventional tool to accurately clarify the etiology of NCD in China and help rapidly employ effective preventive and therapeutic approaches to reduce economic losses.

In summary, we have established a one-step multiplex detection assay for simultaneous detection and characterization of BRV, BCoV, and *E. coli* K99^+^. The method is highly sensitive, specific, and reproducible, which can rapidly recognize the diarrheal pathogens in the clinical samples with mixed infections.

## Data availability statement

The datasets presented in this study can be found in online repositories. The names of the repository/repositories and accession number(s) can be found in the article/supplementary material.

## Author contributions

CW: Data curation, Formal analysis, Investigation, Methodology, Validation, Writing – original draft, Writing – review & editing. FW: Conceptualization, Supervision, Writing – original draft, Writing – review & editing. JC: Methodology, Resources, Writing – review & editing. ZJ: Investigation, Methodology, Validation, Writing – review & editing. YH: Investigation, Methodology, Writing – review & editing. MW: Investigation, Methodology, Writing – review & editing. BJ: Supervision, Writing – review & editing. AZ: Supervision, Writing – review & editing. XY: Conceptualization, Funding acquisition, Supervision, Writing – original draft, Writing – review & editing.
